# Utility of upright radiographs in traumatic thoracolumbar fracture management

**DOI:** 10.1186/s12891-022-05243-7

**Published:** 2022-03-28

**Authors:** Jason Laurita, Jason E. Brant, Kiera Degener-O’Brien, Spencer Smith, Arilene Godoy, Stephanie S. Radoslovich, Jung U. Yoo

**Affiliations:** Department of Orthopaedics and Rehabilitation, Oregon Health & Science University, Sam Jackson Hall, Suite 2360 3181 S.W. Sam Jackson Park Road, 97239 Portland, OR United States of America

**Keywords:** Thoracolumbar, Vertebral, Fracture, Upright radiograph, Kyphosis

## Abstract

**Background:**

It is common practice to use a combination approach of computed tomography (CT) scan followed by upright radiographs when assessing traumatic thoracolumbar (TL) vertebral fractures. The purpose of this study was to determine the clinical utility of upright spine radiographs in the setting of traumatic TL fracture management. Our null hypothesis is that upright TL radiographs rarely change management of acute vertebral fractures.

**Methods:**

A retrospective study was performed on patients with an initial plan of non-operative management for a TL fracture between January 2014 and June 2020 at a single Level 1 trauma center. Patients were followed from time of initial consult to either conversion to surgery (operative) or last available outpatient follow up imaging (non-operative). Lateral kyphotic angle of the fractured vertebra and anterior vertebral body height% loss on initial CT, first upright radiograph, and endpoint upright radiograph imaging were measured. Measurements were compared between and within operative and non-operative groups using t-tests and Mann-Whitney U tests when appropriate. P-values ≤ 0.05 were considered statistically significant.

**Results:**

The study included 70 patients with an average age of 54 years and 37 (52.9%) were women. Six (8.6%) of 70 patients had a change from non-operative to operative management based on upright radiographs. The mean (standard deviation) change in degrees of kyphosis from CT scan to first X-ray was 4.6 (7.0) in the non-operative group and 11.5 (8.1) in the operative group (*P* = 0.03). Delta degrees of kyphosis from CT scan to endpoint X-ray was 6.4 (9.0) and 16.2 (6.2) in the non-operative and operative groups, respectively (*P* = 0.01). In the operative group, mean degrees of kyphosis increased from 1.6 (7.6) in initial CT to 13.1 (8.9) in first X-ray (*P* = 0.02). First X-ray mean anterior body height% loss was 37.5 (17.6) and 53.2 (16.1) in the non-operative and operative groups, respectively (*P* = 0.04).

**Conclusions:**

Upright radiographs are useful in guiding traumatic vertebral fracture management decisions. Larger studies are needed to determine the degree of change in kyphosis between CT and first standing radiograph that is suggestive of operative management.

**Trial registration number and date of registration:**

Not applicable.

## Background

The rate of thoracolumbar (TL) vertebral fractures in blunt trauma has been identified to be 6.90% with the most common causes being attributed to high-energy trauma such as motor vehicle collisions and falls [1]. TL fractures can result in neurological damage, chronic pain, deformity, decreased quality of life, and limitations in activity or return to work [2–4]. Therefore, it is important to appropriately manage these fractures by stratifying them into conservatively managed stable fractures versus unstable fractures requiring surgical stabilization.

TL fractures are often first identified on a computed tomography (CT) scan in the emergency setting. This is because guidelines for the use of a CT scan include polytrauma, high velocity trauma, and suspicion of a vertebral fracture [4–6]. When clinical and imaging findings indicate a stable fracture, these fractures can be treated conservatively with or without bracing whereas unstable fractures are managed surgically. Stability is of primary importance when guiding operative versus non-operative management decisions for vertebral fractures. Mechanical stability of a vertebral fracture is determined by several factors, including the location and orientation of the fracture and the integrity of ligamentous stabilizers of the spinal column such as the posterior ligamentous complex (PLC) [7, 8]. These characteristics can be assessed using both CT and plain radiographic imaging. Signs on imaging such as > 50% decrease in anterior vertebral body height and > 20–30° of kyphotic deformity are suggestive of PLC injury and mechanical instability [7–9]. Of course, imaging is only one component of vertebral fracture assessment and operative versus non-operative management decisions are multifactorial.

There have been various classification systems designed to categorize TL fracture severity and help guide treatment decisions [10–12]. One of the most widely used scoring systems is the Thoracolumbar Injury Classification and Severity Score (TLICS) created by Vaccaro et al. [13]. TLICS is supported as a validated, safe, and effective treatment recommendation for vertebral fractures with good intra- and interrater reliability [14, 15]. The TLICS scoring system can be completed using clinical findings and CT imaging, yet upright radiographs are also used to evaluate fracture stability in certain circumstances.

When a TL fracture appears to be stable/non-operative or indeterminate on CT, it is often interrogated for stability with an upright radiograph with or without the use of a thoracic-lumbar-sacral orthosis (TLSO) brace [5, 9, 16]. This practice gained support after Mehta et al. [17] showed an average increase in anterior vertebral compression of 12% and lateral Cobb angle of 7° between supine and weight-bearing radiographs, which led to a change from non-operative to operative management in 7/28 (25%) of patients. Although using a combination approach of both CT and upright radiographs for these cases is common practice, it is unclear how often these radiographs change clinical management.

The clinical utility of upright radiographs and their effect on the course of treatment for acute TL vertebral fractures based on change of kyphosis and anterior vertebral body height loss between CT and upright radiograph images is evaluated in this study. Our null hypothesis is that using standing radiographs to assess spine stability after CT rarely change the course of treatment (surgical versus non-surgical) and therefore have a low utility. If there is no significant difference between the surgical and non-surgical groups, the elimination of standing radiographs from use in this setting would lead to cost savings and the prevention of needless radiation exposure [18, 19].

## Methods

Approval for a retrospective review of patient records at a single Level 1 trauma center was obtained from our Institutional Review Board. We reviewed all records of patients who presented to the emergency department with acute TL vertebral fractures from January 1, 2014 to June 30, 2020. The review was further limited to patients who had a subsequent Current Procedural Terminology code for a TL radiograph. Upright radiographs included images documented as “standing” and “upright.”

**Fig. 1:**
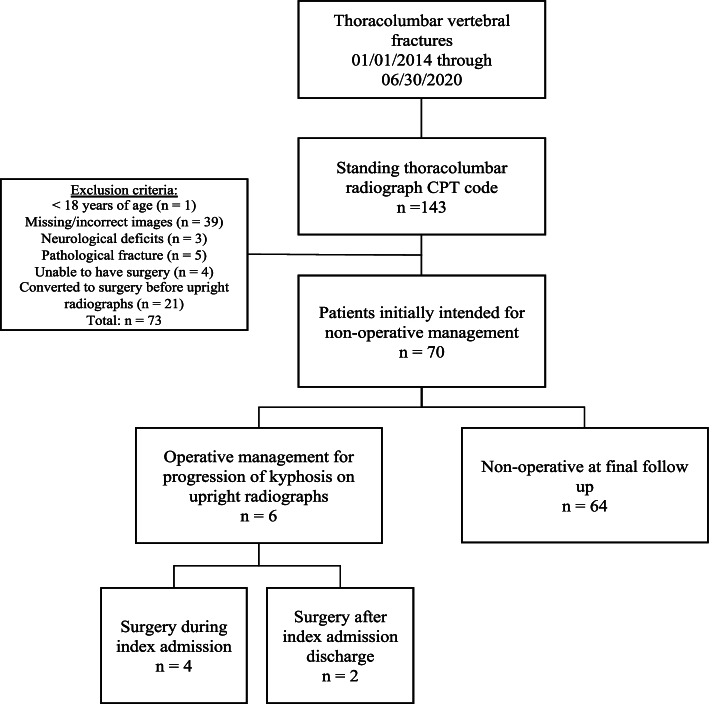
Flowchart of Patients Included

Inclusion criteria consisted of patients ≥ 18 years of age who had a TL fracture, CT scan and subsequent upright radiograph, and an initial plan of non-operative management for their TL fracture after CT scan and before an upright radiograph was taken. All patients in this study received a CT scan as the first imaging modality for their TL fracture around the time of the ED visit or hospital admission day. Exclusion criteria included patients with missing images (CT scan and/or first upright radiograph) or images in the incorrect order (upright radiograph before CT scan), neurological deficits, pathological fractures, and patients who could not have surgery due to medical reasons. Patients with unstable TL vertebral fractures or those who underwent surgery before receiving an upright radiograph were also excluded due to immediate operative fixation at the time of initial consult (Fig. 1).

A detailed chart review was then conducted on the remaining patients who were considered to be initially non-operative. Demographic data and baseline characteristics including age, sex, polytrauma, fracture characteristics (location, type, morphology, multiple fractures), PLC injury, surgeon type (orthopaedic and neurosurgery), and TLSO use were abstracted from patient charts (Table 1). Polytrauma was defined as a patient with another skeletal fracture in addition to a vertebral fracture such as a rib or distal radius fracture. Patient overall TLICS scores at the time of initial consult note were collected from patient charts. The TLICS scoring system is divided into three groups consisting of fracture morphology (1–4 points), neurological involvement (0–3 points), and PCL integrity (0–3 points). These categories are added together for a total score, which recommends non-operative management for ≤ 3 points, non-operative or operative management for 4 points, and operative management for ≥ 5 points [13].

**Table 1:** Patient characteristics

	Total *n* = 70	Non-operative *n* = 64	Operative *n* = 6	*P* Value
**Age**				*P = 0.3954*
Mean (SD)	53.6 (22.3)	52.9 (22.5)	60.3 (20.2)	
Range	18–95	18–95	25–79	
**Sex**				*P = 1*
Female	37	34 (53%)	3 (50%)	
Male	33	30 (47%)	3 (50%)	
**Poly-Trauma**				*P = 1*
No	43	39 (61%)	4 (67%)	
Yes	27	25 (39%)	2 (33%)	
**Fracture Location**				*P = 0.3387*
Thoracic Fracture(s)	31	30 (47%)	1 (17%)	
Lumbar Fracture(s)	29	25 (39%)	4 (67%)	
Thoracic and Lumbar Fractures	10	9 (14%)	1 (17%)	
**Fracture Level**				*P* = 0.3501
T8	1	1 (2%)	0 (0%)	
T9	1	1 (2%)	0 (0%)	
T10	2	2 (3%)	0 (0%)	
T11	5	4 (6%)	1 (17%)	
T12	28	28 (44%)	0 (0%)	
L1	27	22 (34%)	5 (83%)	
L2	5	5 (8%)	0 (0%)	
L3	1	1 (2%)	0 (0%)	
**Multiple Vertebral Fractures**				*P* = 0.6663
No	50	45 (70%)	5 (83%)	
Yes	20	19 (30%)	1 (17%)	
**Fracture Morphology**				*P = 0.3106*
Thoracic Compression	24	24 (38%)	0 (0%)	
Thoracic Burst	11	10 (16%)	1 (17%)	
Thoracic Flexion-Distraction	2	2 (3%)	0 (0%)	
Lumbar Compression	17	15 (23%)	2 (33%)	
Lumbar Burst	16	13 (20%)	3 (50%)	
**TLICS Score**				*P = 0.3893*
1	36	34 (53%)	2 (33%)	
2	16	15 (23%)	1 (17%)	
3	4	4 (6%)	0 (0%)	
4	12	9 (14%)	3 (50%)	
6	1	1 (2%)	0 (0%)	
7	1	1 (2%)	0 (0%)	
**PLC Injury**				*P = 0.2488*
No Injury	53	50 (78%)	3 (50%)	
Indeterminate/Suspected	16	13 (20%)	3 (50%)	
Injured	1	1 (2%)	0 (0%)	
**Consulting Surgeon**				*P = 1*
Orthopaedic	28	26 (41%)	2 (33%)	
Neurosurgery	42	38 (59%)	4 (67%)	
**TLSO in First X-Ray**				***P = 0.028***
No	32	32 (50%)	0 (0%)	
Yes	38	32 (50%)	6 (100%)	
**TLSO in Final X-Ray**				***P = 0.0093***
No	57	55 (86%)	2 (33%)	
Yes	13	9 (14%)	4 (67%)	

Patients were followed from the initial TL fracture consult note to either time of operative fracture fixation or last available outpatient follow up to determine if treatment changed after a standing TL radiograph. Change of treatment was defined as a patient who received operative stabilization of a TL fracture after an upright radiograph was taken. The decision to convert to operative management after a TL upright radiograph was multifactorial and at the discretion of the consulting physician. A total of 16 consulting physicians (9 neurosurgeons and 7 orthopaedic surgeons) were responsible for management decisions including use of a TLSO and conversion to surgery for the patients included in this study. Factors contributing to the operative versus non-operative decision included pain level, comorbidities, social support, increased kyphosis and/or vertebral height loss on upright radiograph when compared to CT scan, PLC integrity, findings on magnetic resonance imaging, and TLICS score. Patients who underwent spine surgery for their TL fracture at any point during the study period were placed in the operative group and those who did not have surgery for their TL fracture were placed in the non-operative group.

**Fig. 2:**
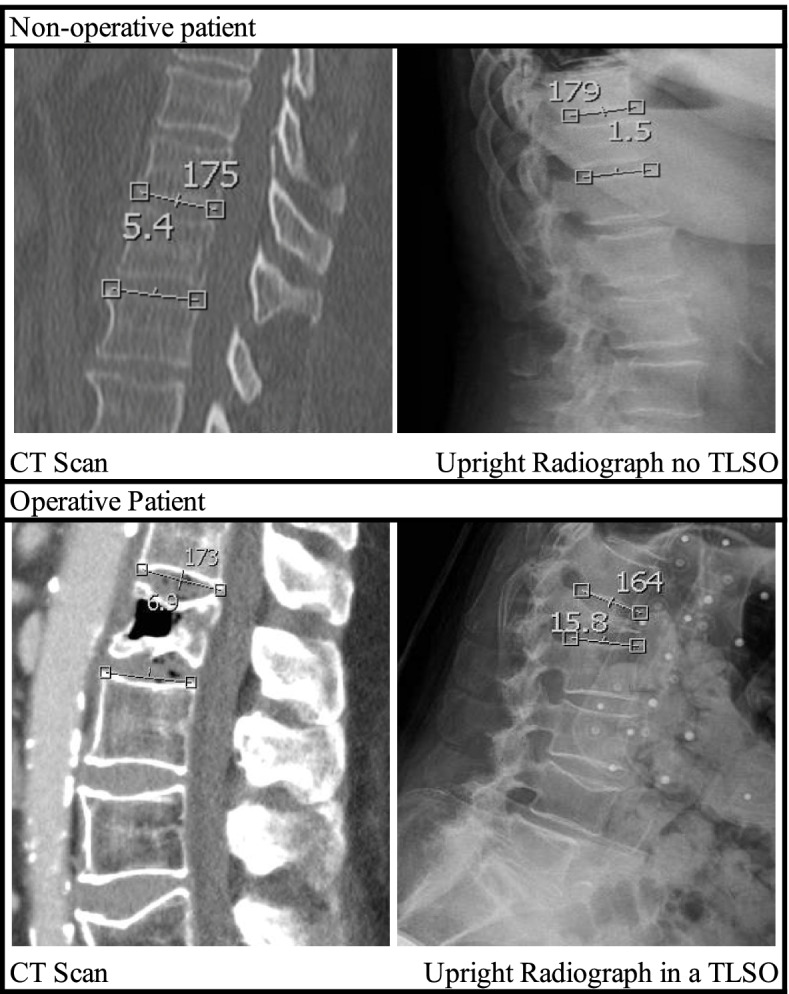
Kyphosis angle measurement

**Fig. 3:**
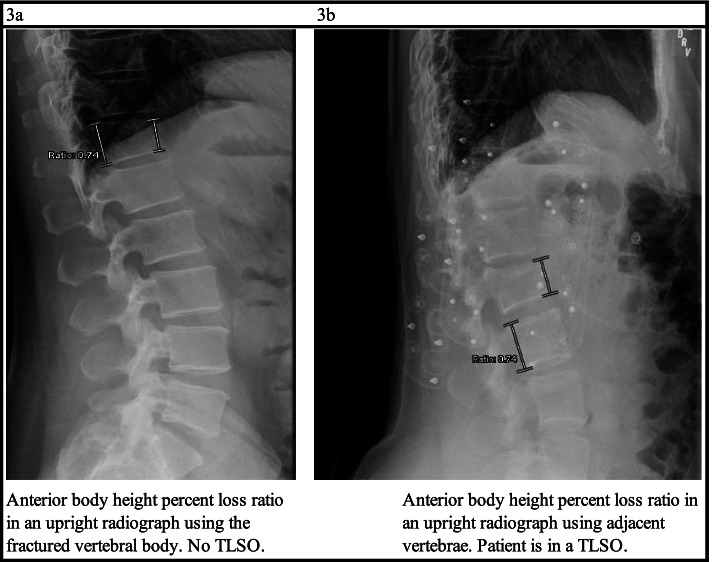
Anterior vertebral body height measurement

PACS imaging software and embedded imagining tools (kyphosis angle, height ratio) were used for all measurements. Degrees of kyphosis through the fractured vertebra and anterior vertebral body height% loss were measured for each patient on all available relevant imaging, which consisted of a midline sagittal CT cut at the time of diagnosis and lateral view upright radiographs on all subsequent imaging. First X-ray was the first upright radiograph taken after initial CT scan during the index admission/emergency department visit. Endpoint X-ray was the final radiograph at the last available outpatient follow up appointment or the final upright radiograph before conversion to surgery. Kyphosis angle was measured from the inferior endplate of the vertebra above the fractured vertebra to the superior endplate of the vertebra below the fractured vertebra. An example of kyphosis measurement in initial CT scan and first upright radiograph for a non-operative and operative patient is shown in Fig. 2. The contrast was adjusted on available imaging until the vertebral body was seen most clearly to obtain as accurate measurement as possible. Anterior vertebral body height% loss was measured as a ratio of the posterior and anterior body heights (Fig. 3a). If the posterior middle column of the vertebral body was affected by the fracture, the posterior body of the vertebrae below the fractured vertebrae was used for the anterior body height loss percent ratio (Fig. 3b). If consecutive vertebrae were fractured, the most severe fracture or vertebral fracture level documented in the consult note was used for kyphotic angle and anterior vertebral body height% loss measurement. Use of TLSO in each upright radiograph was also documented.

Degrees of kyphosis and anterior body height loss were recorded for non-operative and operative groups and compared between and within non-operative and operative groups at three timepoints: initial CT, first standing radiograph during the index admission, and final standing radiograph at endpoint. Additionally, mean change in kyphosis and anterior body height loss from initial CT scan to first upright radiograph and from initial CT scan to final upright radiograph was compared between and within non-operative and operative groups.

Statistical analyses were performed using SAS version 9.4. Categorical variables between non-operative and operative groups were compared using chi-square analysis. When comparing continuous variables between and within non-operative and operative groups, samples were tested for normal distributions and the appropriate analyses were performed. Specifically, independent two sample t-tests were performed on samples with normally distributed values while Mann-Whitney U tests were performed on samples without normally distributed values. P-values ≤ 0.05 were considered statistically significant.

## Results

Initial chart review yielded 143 patients with a TL fracture during the study period. After applying exclusion criteria, 70 patients with an initial non-operative management plan for their TL fracture were found to be eligible for this study. The study included 33 men and 37 women with an average age of 53 and 60 years in the non-operative and operative groups, respectively (Table 1). A total of 37 thoracic and 33 lumbar fractures were examined with morphologies consisting of compression, burst, and flexion-distraction. The most common TLICS score was 1 (53%) and 4 (50%) in the non-operative and operative patients, respectively. For the first upright radiograph, 32 (50%) non-operative patients and 6 (100%) operative patients wore a TLSO brace. TLSO use in the final upright radiograph was 9 (14%) and 4 (67%) in the non-operative and operative groups, respectively. When comparing non-operative and operative groups, there was a significant difference between TLSO use in the first upright radiograph (*P* = 0.03) and in the final upright radiograph (*P* = 0.009). Mean (standard deviation) time in days from CT scan to first X-ray was 8.5 (13.7) and 2.2 (3.1) in the non-operative and operative groups, respectively (*P* = 0.21). The average time in weeks from CT scan to endpoint X-ray was 14.2 (16.3) in the non-operative group and 1.3 (1.7) in the operative group (*P* = 0.001).

Of the included 70 patients, six patients went on to have surgical stabilization of their TL fracture after an upright radiograph. Four of these patients with fracture morphologies of L1 burst (2), L1 compression, and T11 burst underwent surgery during the index admission after their first upright radiograph. The remaining two patients with fracture morphologies of L1 compression and L1 burst had surgical stabilization of their TL fracture after outpatient follow up radiographs demonstrated late fracture subsidence. Factors contributing to the decision to convert to surgery included increased kyphosis or anterior body height loss in first upright radiograph when compared to CT scan and case-specific details such as unbearable pain and discussion with the patient (Table 2). For operative patients, change in kyphosis ranged from 0 to 22.7 degrees and change in anterior vertebral body height loss ranged from − 5 to 53% between CT scan and first upright radiograph (Table 3).

**Table 2:** Description of operative management cases

Converted to Surgery After Outpaitent Follow Up	
**CASE 1**	**CASE 2**
**Age**: 76 years old	**Age**: 50 years old
**Sex**: Female	**Sex**: Male
**Fracture Type**: L1 Compression	**Fracture Type**: L1 Burst
**Polytrauma**: No	**Polytrauma**: No
**TLICS**: 1 (+ 1 for compression fracture)	**TLICS**: 4 (+ 2 burst, + 2 PLC indeterminate)
**Consulting Surgeon**: Neurosurgeon	**Consulting Surgeon**: Neurosurgeon
Factors contributing to operative decision: Endpoint X-ray at one month follow up showed an increase in kyphosis of 24.4 degrees and an increase in anterior body height loss of 45% when compared to first upright X-ray. Patient was having unbearable pain.	Factors contributing to operative decision: Endpoint X-ray at three month follow up showed slight increase in kyphosis of 3.8 degrees and an increase in anterior body height loss of 7% when compared to first upright X-ray. Increased narrowing of T12-L1 disc space and osseous retropulsion on endpoint X-ray.
**Converted to Surgery During Index Admission**	
**CASE 3**	**CASE 4**
**Age**: 79 years old	**Age**: 25 years old
**Sex**: Female	**Sex**: Male
**Fracture Type**: L1 Compression	**Fracture Type**: T11 Burst
**Polytrauma**: No	**Polytrauma**: Yes
**TLICS**: 1 (+ 1 for compression fracture)	**TLICS**: 4 (+ 2 burst, + 2 PLC indeterminate)
**Consulting Surgeon**: Neurosurgeon	**Consulting Surgeon**: Orthopaedic
**Factors contributing to operative decision**: First upright X-ray showed an anterior body height loss of 77%, which was a 46% increase in kyphosis when compared to CT scan. Patient was having difficulties ambulating.	**Factors contributing to operative decision**: Increase in degrees of kyphosis and anterior body height loss in first upright radiograph when compared to CT scan.
**CASE 5**	**CASE 6**
**Age**: 62 years old	**Age**: 70 years old
**Sex**: Female	**Sex**: Male
**Fracture Type**: L1 Burst	**Fracture Type**: L1 Burst
**Polytrauma**: No	**Polytrauma**: Yes
**TLICS**: 4 (+ 2 burst, + 2 PLC indeterminate)	**TLICS**: 2 (+ 2 for burst fracture)
**Consulting Surgeon**: Orthopaedic	**Consulting Surgeon**: Neurosurgeon
**Factors contributing to operative decision**: Increase in degrees of kyphosis, anterior body height loss, and retropulsion (CT − 1 mm, First X-ray − 5 mm) in first upright radiograph when compared to CT scan.	**Factors contributing to operative decision**: Increase in degrees of kyphosis and anterior body height loss in first upright radiograph when compared to CT scan. Discussion with patient.

**Table 3:** Degrees of kyphosis and anterior vertebral body height% loss for operative patients

	Degrees of Kyphosis			Anterior Body Height% Loss		
	**Initial CT**	**First X-ray**	**Endpoint** **X-ray**	**Initial CT**	**First X-ray**	**Endpoint** **X-ray**
**CASE 1**	3.4	3.4	27.8	35%	38%	80%
**CASE 2**	1.6	6.3	10.1	45%	40%	47%
**CASE 3**	-6.9	15.8	*	31%	77%	*
**CASE 4**	14.8	28.7	*	50%	61%	*
**CASE 5**	-4.5	10.9	*	10%	63%	*
**CASE 6**	1.2	13.5	*	32%	40%	*

*For cases 3–6, these patients converted to surgery during their index admission and the first X-ray is the same as the endpoint X-ray

When comparing the non-operative and operative groups, there was no difference in mean degrees of kyphosis at initial CT (*P* = 0.41) and first X-ray (*P* = 0.15). There was a difference between non-operative and operative groups in mean degrees of kyphosis at the endpoint X-ray. The mean (standard deviation) at endpoint X-ray was 9.5 (11.8) and 17.8 (8.3) for the non-operative and operative groups, respectively (*P* = 0.02). Change in degrees of kyphosis from CT scan to first X-ray was 4.6 (7.0) in the non-operative group and 11.5 (8.1) in the operative group (*P* = 0.03). Delta degrees of kyphosis from CT scan to endpoint X-ray was 6.4 (9.0) and 16.2 (6.2) in the non-operative and operative groups, respectively (*P* = 0.01). Mean anterior body height% loss was 37.5 (17.6) in the non-operative group and 53.2 (16.1) in the operative group at the first X-ray (*P* = 0.04). At the endpoint X-ray, mean anterior body height% loss was 42.5 (19.7) and 61.3 (15.9) in the non-operative and operative groups, respectively (*P* = 0.02). There was no difference between non-operative and operative groups in change in anterior body height% loss from CT scan to first X-ray (*P* = 0.34) and from CT scan to endpoint X-ray (*P* = 0.10) (Table 4).

**Table 4:** Mean values between treatment groups at all imaging time points

	Non-operative	Operative	*P V*alue
**Degrees of Kyphosis**			
Initial CT (SD)	3.4 (8.0)	1.6 (7.6)	*P* = 0.4122
First X-Ray (SD)	8 (10.2)	13.1 (8.9)	*P* = 0.1536
Endpoint X-Ray (SD)	9.5 (11.8)	17.8 (8.3)	***P*** **= 0.0216**
**Delta Degrees of Kyphosis from CT**			
First X-Ray (SD)	4.6 (7.0)	11.5 (8.1)	***P*** **= 0.0259**
Endpoint X-Ray (SD)	6.4 (9.0)	16.2 (6.2)	***P*** **= 0.0097**
**Anterior Body Height% Loss (%)**			
Initial CT (SD)	28.3 (16.1)	33.8 (13.9)	*P* = 0.1931
First X-Ray (SD)	37.5 (17.6)	53.2 (16.1)	***P*** **= 0.0449**
Endpoint X-Ray (SD)	42.5 (19.7)	61.3 (15.9)	***P*** **= 0.0232**
**Delta Anterior Body Height% Loss from CT (%)**			
First X-Ray (SD)	9.3 (12.3)	19.3 (24.1)	*P* = 0.3388
Endpoint X-Ray (SD)	13.5 (17.5)	27.5 (22.8)	*P* = 0.1037

Degrees of kyphosis and anterior body height% loss both significantly increased when comparing initial CT to first X-ray in the non-operative group. The mean (standard deviation) degrees of kyphosis in initial CT scan 3.4 (8.0) differed from 8 (10.2) in the first X-ray (*P* < 0.001). Anterior body height% loss was 28.3 (16.1) and 37.5 (17.6) in the initial CT and first X-ray, respectively (*P* < 0.001). In the operative group, degrees of kyphosis increased from 1.6 (7.6) in initial CT to 13.1 (8.9) in first X-ray (*P* = 0.02). Both non-operative and operative groups had an increase in degrees of kyphosis and anterior body height% loss when comparing initial CT scan and endpoint X-ray measurements between individual treatment groups. The increase in degrees of kyphosis between initial CT 1.6 (7.58) and endpoint X-ray 17.8 (8.34) within the operative group was the only comparison of this type to be non-significant (*P* = 0.29) (Table 5).

**Table 5:** Mean values within individual treatment groups from initial CT to first X-ray (A) and from initial CT to endpoint X-ray (B)

A	Initial CT	First X-Ray	*P* Value
**Kyphosis Degrees (Degrees)**			
Nonoperative (SD)	3.4 (8.0)	8 (10.2)	***P*** **< 0.001**
Operative (SD)	1.6 (7.6)	13.1 (8.9)	***P*** **= 0.0175**
**Anterior Body Height% Loss (%)**			
Nonoperative (SD)	28.3 (16.1)	37.5 (17.6)	***P*** **< 0.001**
Operative (SD)	33.8 (13.9)	53.2 (16.1)	*P* = 0.1065
**B**	**Initial CT**	**Endpoint** **X-Ray**	***P*** **Value**
**Kyphosis Degrees (Degrees)**			
Nonoperative (SD)	3.39 (8.08)	9.5 (11.8)	***P*** **< 0.001**
Operative (SD)	1.6 (7.58)	17.8 (8.34)	*P* = 0.29
**Anterior Body Height% Loss (%)**			
Nonoperative (SD)	28.3 (16.1)	42.5 (19.7)	***P*** **< 0.001**
Operative (SD)	33.8 (13.9)	61.3 (15.9)	***P*** **= 0.0318**

## Discussion

The decision of whether to treat a TL vertebral fracture either conservatively or with surgery can be challenging. The validated TLICS scoring system is an objective and effective tool for guiding operative versus non-operative decisions for management of vertebral fractures; however, a score of 4 is indeterminate [20]. The decision to proceed to surgery for vertebral fractures with indeterminate stability (TLICS score 4) and potentially stable fractures (TLICS score ≤ 3) is affected by a combination of several different factors. Radiographic factors that are used to guide management decisions include kyphotic angle, severity of vertebral fracture, vertebral body height loss, fracture location, integrity of posterior column structures, and remaining canal area [7, 21]. When the TLICS score is indeterminate or there is cause for uncertainty as to whether surgery is indicated for instability, standing radiographs of the TL spine are taken at 1, 3, 6, and 12 weeks to monitor the healing process and identify occult instability not recognized on initial evaluation [5].

As shown in this study, monitoring of vertebral fractures with sequential upright radiographs can lead to a change from conservative to surgical management. However, vertebral fracture management guidelines are inconsistent and there is no general consensus as to what factor is weighted more when selecting the appropriate treatment plan [22, 23]. This is why the same type of fracture and/or TLICS score can have different treatment paths depending patient injury and/or circumstances. In this study there were a total of nine neurosurgeons and seven orthopaedic surgeons who were involved in vertebral fracture management decisions. There could be differences in training and practices, but there were comparable amounts of neurosurgeons and orthopaedic surgeons in the non-operative and operative groups. In this study, it was discovered that decisions to follow conservative or operative pathways were based on findings on upright radiographs and patient situations. Two patients in this study with TLICS scores of 6 and 7 had flexion-distraction fractures, which would typically suggest surgery due to a higher degree of injury. However, both were treated conservatively with a TLSO brace. Despite having an injured PLC, the first patient had limited social support, active IV drug use, and poor nutritional status, which did not make them a good candidate for surgery. The second patient had an indeterminate PLC injury, but was determined to have a stable fracture after the first upright radiograph confirmed fracture stability. This decision to manage conservatively was supported by the anterior body height loss being 21% on initial CT scan and 16% on endpoint X-ray at the 3 month follow up. Additionally, the kyphotic increase from CT scan to endpoint X-ray was only 0.8 degrees. This is an example of how upright radiographs can be useful in guiding vertebral fracture management decisions and prevent patients from having unnecessary surgery and the risks associated with it.

In this study, the effectiveness of upright radiographs as a tool to guide operative versus non-operative decisions for traumatic vertebral fracture management was evaluated. Standing radiographs led to a change of plan from presumed non-operative to operative treatment in 6 (8.6%) of 70 patients. Four of the six operative patients converted to surgery during the index admission after showing an increase in kyphosis ranging from 12.3 to 22.7 degrees and an increase an anterior body height loss ranging from 8 to 53% when comparing initial CT scan to first upright radiograph. These patients had acute fractures and the time from initial CT scan to first X-ray ranged from 0 to 8 days. This suggests that factors such as age or undiagnosed osteopenia did not play a role in the fracture subsidence. Instead, findings on first upright radiographs such as increased kyphosis compared to CT scan and average anterior body height loss guided management decisions. There was a significant difference between non-operative (38%) and operative (53%) anterior body height loss in this study. This amount of anterior body height loss in the operative group is similar to literature suggesting > 50% loss of height indicates mechanical instability and the need for surgery [8]. Within the operative group, the increase in kyphosis on first X-ray (13 degrees) compared to initial CT scan (2 degrees) was found to be statistically significant. Patients in this study had a conversion to surgery at a ~ 11 degree increase in kyphosis from CT scan to first X-ray and an average 13 degrees of kyphosis on first upright radiograph. This degree of kyphosis is lower than the > 30 degrees that has been reported as a level of kyphosis indicating instability and likely need for surgical management [8]. Our results illustrate how physicians in clinical practice may decide to operate at a lower increase in kyphotic threshold than is suggested. However, there is no universally agreed upon angle of kyphosis that warrants surgical management and there is conflicting evidence about whether or not increased kyphosis is predictive of conservative management failure [21].

Alimohammadi et al. [25] suggested that there is a possible relationship between increased kyphosis and failure of conservative therapy for vertebral fractures. However, this study focused on only burst fractures and compared kyphosis on all available imaging (CT, X-ray, MRI) instead of specifically upright radiographs. We assessed whether the degree of kyphotic angulation through the fractured vertebra on the first upright radiograph had a correlation with the decision to proceed to surgical stabilization. There was a significant difference in the degree of kyphotic collapse on the first standing radiograph in operative patients (~ 12 degrees) versus non-operative patients (~ 5 degrees). Additionally, in the first upright radiograph, there was a significant difference in anterior body height loss of 38% and 53% between the non-operative and operative groups, respectively. This supports that an increase in kyphosis from CT scan to the first upright radiograph could be relevant in fracture management decision making processes, but larger studies are needed to confirm this.

There is no general consensus on the use of a TLSO for TL fractures. Research has shown that there is no significant difference in functional outcomes and kyphosis for TL fracture patients treated with or without a TLSO [24]. This could explain why there was variable use of a TLSO in radiographs for the non-operative patients in this study. However, 50% of non-operative patients had their first upright radiograph in a TLSO. In the operative group, five of six patients who converted to surgical management had kyphotic collapse in their first upright radiograph when compared to initial CT scan despite the use of a TLSO. Additionally, the two patients who underwent surgical stabilization after outpatient follow up did so only after their first upright radiograph without a TLSO, which showed a further progression of kyphosis. TLSO use both allowed and hid kyphosis progression on upright radiographs for the operative patients in this study, therefore, it is yet to be determined if and/or how TLSO use affects markers of stability on upright radiographic imaging.

There were limitations in this study. First, this was a retrospective study, which is prone to confounding. Data was collected for all eligible patients in an attempt to mitigate selection bias, though factors that could influence measurements such as BMI and smoking were unable to be abstracted from all patient charts. However, four of the six patients who converted to surgery during their index admission did so shortly after an upright radiograph, which suggests that factors influencing the conversion to surgery were more acute and related to the increase in kyphosis seen on first X-ray. Additionally, patients with neurological injury were eliminated from this study because acute change in neurologic function could be an indication for surgery regardless of radiological findings. Eliminating patients with things that could influence management substantially (pathological fracture, neurological injury) further supports the association between imaging measurements and surgical decisions. Second, upright radiographs are vulnerable to slight differences in patient position and image quality (imaging blurs), which could affect measurement values. Measurements were taken at specific points for each image and steps were taken including adjusting image contrast to best visual measurement points and having measurements checked for consistency. The slight differences in measurements that could occur would be identical to that seen in clinical practice, which supports the application of this study. Third, the sample size in this study is small. Larger multicenter studies with the inclusion of relevant confounding factors that could affect traumatic TL fracture measurements and management decisions are necessary.

## Conclusions

Upright TL radiographs can be used to guide treatment decisions for vertebral fractures of indeterminate stability. This research suggests that there is a significant difference in kyphotic progression through the vertebral fracture of non-operative versus operative fractures.

## Data Availability

The datasets used for this study are available from the corresponding author on reasonable request.

## Abbreviations

TL: Thoracolumbar
CT: Computed tomography
PLC: Posterior ligamentous complex
TLICS: Thoracolumbar injury classification and severity score
TLSO: Thoracic-lumbar-sacral orthosis
